# Therapeutic utility of Lung-MAP: ushering into an era of genomic and biomarker-driven clinical trials

**DOI:** 10.1038/s41392-021-00557-9

**Published:** 2021-04-01

**Authors:** Chenyue Zhang, Haiyong Wang

**Affiliations:** 1 Department of Integrated Therapy, Fudan University Shanghai Cancer Center, Shanghai Medical College, Shanghai, China; 2 Department of Internal Medicine Oncology, Shandong Cancer Hospital and Institute, Shandong First Medical University and Shandong Academy of Medical Sciences, Jinan, China

**Keywords:** Lung cancer, Genome

In a recent article published in Lancet Oncology, Redman^1^ demonstrated us The Lung Cancer Master Protocol (Lung-MAP; S1400) in a detailed manner. The authors introduced us screening registrations, assignments, and sub-study registrations carefully.

**Fig. 1:**
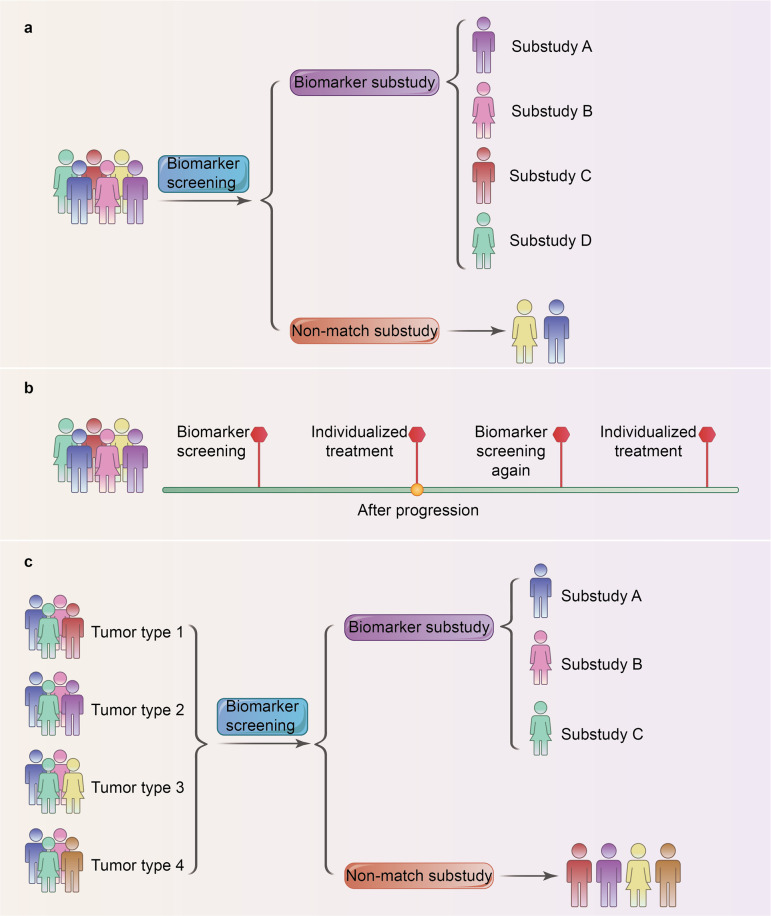
Clinical trial design flow chart to guide individualized treatment. **a** The schema demonstrates the protocol of the umbrella study. Eligible patients of a specific cancer type are assigned to sub-studies based on their biomarkers or to a “nonmatch” sub-study if the patient does not qualify for the biomarker-specific sub-studies. **b** Patients are assigned to individualized treatments according to their biomarkers. Treatment continues within each sub-study until tumor progression has been detected. Patients would be reassigned based on biomarker screening again. This is a repeated process. **c** The schema demonstrates the protocol of the basket study. Patients with different cancer types are assigned to sub-studies based on their biomarkers. They would undergo a targeted therapy directed against a specific genetic mutation even in different cancer types

Unlike conventional clinical trials, Lung-MAP study is a biomarker-driven umbrella trial testing the effect of different drugs on different mutations for squamous cell lung cancer (LUSC) (Fig. 1a). It is known that LUSC is intimately associated with tobacco exposure and could possibly amount to a high tumor mutation burden.^2^ Different subtypes of LUSC have been defined according to comprehensive analyses of genomic alterations.

In this trial, LUSC patients underwent genomic testing by next-generation sequencing (NGS). These molecular markers in Lung-MAP study are defined by specific genomic alterations, as demonstrated in different forms of mutations, amplifications and rearrangements. Amounts of studies have reported on the dysregulation of PI3K/AKT, CDK4/6, and FGFR pathways in many cancer types.^3–5^ Aberration in PI3K-AKT pathway has involved in the tumorigenesis in many types of cancer. Inhibition of CDK4/6 pathway has demonstrated favorable effects in patients with dysregulated CDK4/6 activity. Anti-FGFR compounds are also showing promising results in the treatment of many cancers.

As described in the article, the LUSC patients were assigned to biomarker-specific sub-studies where an investigational targeted agent would be compared to standard treatments according to genomic profiling. The sub-studies evaluated the newly developed drugs based on the following mutations: (1) taselisib for PIK3CA mutations, (2) palbociclib for CDK gene alteration, (3) AZD4547 for FGFR gene alteration, (4) rilotumumab against MET plus erlotinib targeting EGFR, (5) talazoparib targeting PARP and telisotuzumab vedotin against MET. Non-match sub-studies evaluated durvalumab and nivolumab plus ipilimumab versus nivolumab for anti-PD-1/PD-L1 naive disease, and durvalumab and tremelimumab for anti-PD-1/PD-L1 relapsed or refractory disease.

In Lung-MAP trial, if patients were found to harbor PIK3CA mutation, they would be assigned to S1400B sub-study. As the study has revealed, S1400B was closed at interim analysis for futility. For patients harboring mutations in CCND1, CCND2, CCND3, and CDK4, they would be allocated into sub-study S1400C, subjecting to palbociclib (investigational) vs docetaxel (standard of care). However, this sub-study failed to demonstrate the pre-expected criteria for moving to the phase III trial. The S1400D sub-study evaluating AZD4547 (investigational) vs docetaxel (standard of care) had minimal activity but bearable safety in LUSC patients with predominant FGFR1/FGFR3-amplification.

Next, the authors have defined both the primary endpoint and secondary endpoints, as well as when should be moved to the next phase III section and when the sub-study should be closed. A comparison of progression-free survival (PFS) between the experimental and standard drug in each group has been made. Endpoints would be overall survival (OS) and PFS if a drug was found to gain a superior position in phase II, thus moving to phase III. Secondary endpoints included safety and toxicity evaluation. Insufficient activity at interim analysis and discontinuation of drug development would result in the ending of sub-studies.

Among the sub-studies of Lung-MAP, some have not reached the endpoints and some have yielded negative results. The following reasons could be possible: Mere mutations alone are not sufficient for the treatment of patients, and epigenetic factors important for gene expression and function are also necessary. Therefore, the determination of aberrations as dominant oncogenes or trunk events should be further validated by functional studies. Rather, some of the trunk events may exert function in tumor development and progression and their inhibition would be tested to be beneficial for cancer patients.

Considering the futility of some sub-studies, the Lung-MAP trial has made some flexible and adaptable changes. For instance, the drug and biomarker selection has been transformed into a fluid process, during which new targeted drugs would replace the existing drugs upon tumor progression, as tested by biomarker screening again (Fig. 1b). Besides, the criterion for included patients has also been redefined. Patients receiving more systemic therapies have access to the trial. The adaptability of the framework not only encourages the introduction of new efficient drugs, but also includes the number of eligible patients to the fullest.

Despite far from expectation, the Lung-MAP study warrants our appraisal and appreciation as the first lung cancer “umbrella study”, which ushers into an era of umbrella clinical trials. It is in contrast to “basket studies” evaluating the effect of a specific drug on a defined molecular target in a variety of cancer types (Fig. 1c). The two innovative clinical trial designs of umbrella test and basket test may reflect the concept of “treating the same disease with different methods” and “treating different diseases with the same method”. Moreover, Lung-MAP has provided us with a platform upon which bountiful resources of blood, tissue, radiology and survival of LUSC patients could be acquired. Further analysis of this information of each individual patient would move a step forward into promoting personalized therapy. Meanwhile, we should note that the Lung-MAP undertaking is by no means an effortless one. The design, implementation and completion require strenuous efforts from all walks of like such as the National Cancer Institute, pharmaceutical companies, Food and Drug Administration and medical professionals.

The Lung-MAP trial has brought us in-depth thinking about the prospective biomarker-driven studies in the genomic era. Undeniably, some lessons should be drawn from the study. True driver mutations should be distinguished from branch or passenger mutations, otherwise, distinct advantages incurred by its biomarker-propelled nature could not be fully embodied. Therefore, understanding of mutational type would be much appreciated by the employment of up-to-date technologies. Moreover, the development of drugs targeting dominant mutations are much expected. However, genetic alterations in LUSC are both complicated and heterogeneous, which renders the development of targeted drugs difficult in LUSC.

In summary, this study has highlighted the Lung-MAP’s design and scheme, eligibility criteria, opportunities and challenges. Importantly, the Lung-MAP study brought targeted therapies to LUSC patients by undertaking a novel clinical trial design via clustering backbones from all industries. This study may perfectly serve as a paradigm for clinical trials laying ahead in heterogeneous cancers.
